# Individuals’ Power Persistence in Teams: A Study Examining the Effects of Individuals’ Competence, Uncooperative Behavior and Team Performance in the National Basketball Association

**DOI:** 10.3389/fpsyg.2022.813346

**Published:** 2022-03-09

**Authors:** Constantinos S. Mammassis, Petra C. Schmid

**Affiliations:** ^1^Department of Business Administration, Athens University of Economics and Business, Athens, Greece; ^2^ETH Zürich, Zürich, Switzerland

**Keywords:** power, power persistence, power dynamics, NBA teams, ordered probit models

## Abstract

This paper examined (a) the persistence of individuals’ power in teams and (b) the individual- and team-level factors influencing power maintenance and loss in the long-term (i.e., power dynamics). Specifically, and in line with the functional theory of power, we showed that individuals’ state of power in the past exerted a significant behavioral impact on their later state of power, hence, confirming the “power persistence” hypothesis. Furthermore, and in accordance with the conflict theory of power, we found that individuals’ competence positively influenced power above and beyond its persistence. We also showed that individuals’ uncooperative behavior and team performance had a negative and significant effect on individuals’ power above and beyond its persistence. Finally, we discussed the importance of individuals’ power dynamics for effectively managing power struggles in teams.

## Introduction

Power is inherent in economic, political, and social interactions influencing the behavior and performance of individuals, teams, and organizations (Keltner et al., 2003; Magee and Galinsky, 2008; Anderson and Brion, 2014; Sturm and Antonakis, 2015; Guinote, 2017). *Power* is defined as the asymmetric control over valued resources, which in turn affords an individual the ability to control others’ outcomes, experiences, or behaviors (Emerson, 1962; Keltner et al., 2003; Magee and Galinsky, 2008; Anderson and Brion, 2014; Tost, 2015). While scholars recently suggested that power should be primarily viewed as “having the discretion and means to enforce one’s will over others” (Sturm and Antonakis, 2015, p. 139) we concur with Tost (2015, p. 31) that “the one (i.e., control over resources) begets the other (i.e., ability to enforce one’s will).” Thus, we consider power as relative, allowing us to explore how the dynamics of having power over others change over time (Tost, 2015).

Although an increasing number of scholars are studying the outcomes of power, little is yet known about the dynamics of power in organizational settings (Magee and Galinsky, 2008; Anderson and Brion, 2014; Sturm and Antonakis, 2015). The functionalist theory of power assumes that power is mostly static and self-sustaining (Magee and Galinsky, 2008; Tarakci et al., 2016). On the other hand, the conflict theory of power suggests that individuals’ power can and does change (Greer and van Kleef, 2010; Sturm and Antonakis, 2015; Greer et al., 2017). Two seminal reviews on individuals’ power highlighted the factors that may lead to the development, maintenance, and loss of power (Anderson and Brion, 2014), and suggested a variety of variables that may facilitate or hinder the persistence of power (Magee and Galinsky, 2008). Both of these papers called for more longitudinal research revealing the dynamics of power in applied settings as most of the research reviewed was based on cross-sectional studies performed in laboratories. In a notable exception, Tarakci et al. (2016) performed a simulation study followed by two surveys and showed that power may be both static and dynamic suggesting that both functional- and conflict-based views of power should be integrated. However, extant literature has generally failed to reconcile these conflicting theories (i.e., functional and conflict) because of a dearth in empirical research tackling how individuals may gain, maintain, and lose power in complex organizational settings over time (Anderson and Brion, 2014; Sturm and Antonakis, 2015).

This paper is, therefore, structured around the following research question: “*How can individuals gain power in team settings and how this power can be maintained and/or lost in the long-term*?.” To respond to this question, and in line with the functional theory of power (Magee and Galinsky, 2008), we first examined the core characteristic of power, its self-reinforcing nature. Specifically, we investigated the hypothesis that power *persists* (i.e., that there is genuine state dependence between an earlier and a later state of power) over time, which is a well-known assertion that has not, yet, been empirically examined longitudinally.

Second, focusing on the conflict theory of power (e.g., Tarakci et al., 2016; Greer et al., 2017), which sees power as a *changeable* state, we explored the individual and team-level factors contributing to the persistence (or loss) of individuals’ power above and beyond its self-reinforcing nature. This extends current research by exploring the nested data structure of power relations in individuals, teams, and performance episodes. To do so, we employed advanced econometric techniques (i.e., random effects ordered probit models), which accounted for both individuals observed and unobserved heterogeneity in our analyses. This approach allowed us to examine individuals’ power as a function of variables that change over time, which in turn change individuals’ power over time. As such, we were able to generate novel evidence on the role of individuals’ time-variant and invariant characteristics to the acquisition and maintenance of power in teams.

To summarize, with this research, we made three contributions to the literature of power in teams. First, we reconciled the functionalist and conflict perspectives of power by considering and integrating both the static and dynamic elements of individuals’ power (Magee and Galinsky, 2008; Greer and van Kleef, 2010; Sturm and Antonakis, 2015). Our econometric models also incorporated the fact that some individuals’ “…power changes dynamically and others’ remain unchanged at the same time…,” thus, responding to recent calls for more research in this field (Tarakci et al., 2016, p. 426). Second, we investigated the determinants of power persistence in a longitudinal model that incorporated several individual (age, race, competence, etc.) and team-level (tenure, performance, etc.) factors that may influence individuals’ power. We explored how these factors play out both for gaining and maintaining power, specifically, when power persistence was taken into account. Hence, we expanded previous research by responding to calls for investigating how varying degrees of power, the tenure of the powerholder, and/or the intrinsic desire to retain power influence power dynamics more generally (Anderson and Brion, 2014; Sturm and Antonakis, 2015). Third and more broadly, we provided new and important insights into how power dynamics in teams emerged, which in turn affected individuals’ contributions toward team functioning and outcomes (Call et al., 2015; Kehoe et al., 2018), as well as on how power struggles within teams should be managed (Hildreth and Anderson, 2016; Tarakci et al., 2016; Greer et al., 2017). Overall, the importance of examining this nested data structure of power relations lies at the fact that extending our knowledge of the exact factors that go beyond power’s persistence may help managers to alter power hierarchies from stable to dynamic and vice versa when necessary (e.g., Magee and Galinsky, 2008).

## Theory and Hypotheses

### Power Persistence

Power is a fundamental characteristic of human relationships and has been extensively researched over the years (Kipnis, 1972; Marx, 1844/1964; for a recent review see Sturm and Antonakis, 2015). Management scholars have recently shown an increased interest in the nature of power, its properties, and its outcomes especially in relation to group functioning and performance (Hildreth and Anderson, 2016; Tarakci et al., 2016; Greer et al., 2017). Seminal reviews have also reinstated the interest in individual power and how it may be gained, maintained, and lost (Keltner et al., 2003; Anderson and Brion, 2014; Sturm and Antonakis, 2015).

Critical questions in the research regarding the nature of individuals’ power in teams are the following: Is power self-reinforcing? Do powerful people always remain powerful? If yes, how? How may powerless people attain high power? Anderson and Brion (2014) devoted a large part of their review theorizing about the factors that affect individuals’ maintenance (or loss) of power while Magee and Galinsky (2008) theorized about the nature of power and, specifically, about how power may reinforce itself. Drawing (mostly) on social psychological research that manipulated power, they concluded that “power begets power as individuals accumulate more valued resources” (Magee and Galinsky, 2008, p. 371). So far, however, only theoretical claims have been made to support the notion that power persists. These theoretical claims are best tested within longitudinal studies; however, longitudinal designs in power research have been extremely scarce (Sturm and Antonakis, 2015). Using such a longitudinal design, we will test the prediction of the functional theory of power that power is self-reinforcing and thus, that there is an important effect between an earlier state of power and a later state of power (i.e., there is genuine state dependence). Hence, we hypothesize that as:

*Hypothesis 1*: Power is persistent over time; that is, individuals who are powerful at *t*−1 have a higher probability to be powerful at *t* = 1.

### Power Maintenance and Power Loss (Above and Beyond Its Persistence)

If power was simply reinforcing itself, one would expect that the powerful would always remain powerful while the powerless would have no or little possibilities to increase their power over time. In contrast, the conflict theory of power suggests that power structures can and do change (e.g., Tarakci et al., 2016; Greer et al., 2017). Several factors have been identified that may bestow power to an individual, help him maintain it, or lose it (for an overview, see: Keltner et al., 2003; Anderson and Brion, 2014; Sturm and Antonakis, 2015). Our purpose here is not to repeat the extant reviews but to identify a few “key” factors that may affect individuals’ power in team settings above and beyond its persistence, in order to demonstrate *how* power may be maintained or lost over time. We focus on three “key” factors that may affect power persistence above and beyond its self-reinforcing nature that are either endogenous (i.e., within the individual powerholder) or exogenous (i.e., operating outside the individual powerholder) following Anderson and Brion’s (2014) definitions.

First, focusing on an endogenous factor, we expect individuals’ *competence* to positively influence their power above and beyond its persistence. Previous research has established a strong and positive association between competence and power (Treadway et al., 2013; Anderson and Brion, 2014). As Guinote (2017, p. 361) notes “…power is readily conferred to individuals who have visible skills or attributes that contribute (or appear to contribute) to the solution of group problems….” Therefore, the more the individuals contribute to their teams, the more their power should be enhanced *beyond* its persistence. In line with the above arguments, our hypothesis reads as:

*Hypothesis 2*: Individuals’ competence (i.e., contribution to their teams) positively affects power over and above its persistence.

Second, we argue that individuals’ *uncooperative behavior*, as another endogenous factor, negatively affects their power above and beyond its persistence. Previous research has shown that when high-power individuals felt that their power may not be stable over time, they experienced more stress (Sapolsky, 2005) and used their resources more toward serving their own (rather than their teams’) interests (Bendersky and Shah, 2012; Mead and Maner, 2012), suggesting that power instability promotes self-serving behavior. In contrast, Kim et al. (2017) found that powerful individuals showed more unethical behavior only when power was stable, but not when it was unstable. In highly competitive teams, however, where the input of an individual directly affects the output of the other team members (i.e., high levels of reciprocal interdependence; Chen and Garg, 2018), high-power individuals’ uncooperative behaviors are expected to negatively influence the persistence of their power. Hence, we posit that as:

*Hypothesis 3*: Individuals’ uncooperative behavior negatively affects power over and above its persistence.

Third, focusing on an exogenous factor, we expect that the *team performance* will negatively affect individuals’ power persistence. Previous research has shown that, in very competitive teams, powerholders face very demanding environmental conditions, which make it challenging to develop or hold into their power (Anderson and Brion, 2014). This is because severe team competitiveness makes low-power individuals strive for the resources held by their high-power counterparts (Fleming and Spicer, 2008) and the powerholders thus risk to lose control over the available resources. Another reason is that high-power individuals receive more social attention and may be incorrectly blamed for any negative team outcome (Sutton and Galunic, 1996), which may also result in a loss of power. Furthermore, in highly competitive teams (compared to less competitive teams), high-power individuals are more likely to lose power as these teams experience higher levels of process conflict that may reduce their performance (Greer et al., 2011). Therefore, we hypothesize that as:

*Hypothesis 4*: Team performance negatively affects individuals’ power over and above its persistence.

## Methods

### Research Context: The National Basketball Association

To test our hypotheses, we turned to the NBA context. Scholars theorizing about organizational phenomena often use basketball as a research context because it represents a complex and uncertain environment in which individual and team-level characteristics can be objectively measured (Christie and Barling, 2010; Ethiraj and Garg, 2012; Halevy et al., 2012; Ertug and Castellucci, 2013; Smith and Hou, 2014; Zhang, 2017; Chen and Garg, 2018). In terms of our research, the NBA context is appropriate to test our hypotheses for at least four reasons. First, NBA teams are characterized by high levels of reciprocal interdependence (Chen and Garg, 2018), which allows for certain individuals to continuously gain and lose power, thus, allowing us to study the notion of power persistence (Hypothesis 1). Second, fine-grained individual data allow us to measure individuals’ competence as contribution to team’s outputs (Hypothesis 2) and to use team-level data to measure team performance (Hypothesis 4; Ethiraj and Garg, 2012; Smith and Hou, 2014). Third, players in the NBA can repeatedly choose whether to cooperate or not (Halevy et al., 2012) and as such uncooperative behavior is particularly salient in NBA teams (Christie and Barling, 2010; relevant to our Hypothesis 3). Fourth, power structures in NBA teams are well defined as cross-memberships do not exist (i.e., one player cannot be part of two teams at the same time).

### Data Sources

The majority of our data came from Basketball Reference,^1^ which is a leading source of NBA data used by organizational scholars and endorsed by experts in NBA analytics (Ethiraj and Garg, 2012; Smith and Hou, 2014; Chen and Garg, 2018). Data on ejections from play and suspensions, which are available from 1997 onward, came from Patricia Bender^2^ and Doug Stats (Ethiraj and Garg, 2012).^3^ Data on injuries came from Pro Sports Transactions (Chen and Garg, 2018).^4^ We also utilized several other sources to compensate for missing data such as data on wages from Rodney Fort^5^ and Forbes.^6^ Our usable sample covers 8,383 player-years from 1997 to 2015. However, some of the independent variables computed were based on data going back to 1994 (this is indicated in the description of each variable).

### Measures

#### Dependent Variable

##### Power

To develop a measure of individuals’ power, we relied on the definition of power as control over critical resources (Magee and Galinsky, 2008; Anderson and Brion, 2014; Tost, 2015) and the broader research on the measurement of individual’s power in organizational settings (e.g., Finkelstein, 1992; Cannella and Shen, 2001; Krause et al., 2015). Since, in the NBA, players are nested within teams, their power can best be captured through the amount of team’s critical resources they control. Previous research has measured individual’s power (i.e., critical resources held) in organizational settings through composite variables accounting for formal power, expertise, structural power, and prestige (see, for instance, Finkelstein, 1992). Similarly, and to measure NBA players’ power in a given season, we consider the following variables:

First, the salary of each player as a proxy for formal power. Since 1984–1985, the NBA league defines a maximum amount of resources that each team can spent on players’ salaries (i.e., salary cup). Salary is a direct indicator of a player’s resource control within his team, as the higher salary a player gets the less amount of money (resources) remains for his teammates. Previous research has also considered salary as a proxy for individual’s formal power within organizations (e.g., Finkelstein, 1992; Zajac and Westphal, 1996; Krause et al., 2015). Thus, greater salary indicates greater power.

Second, the tenure of the player in the league as a proxy for expertise. Individuals with longer tenure are more likely to have social power (Mehra et al., 2001) and carry valuable informal knowledge (Rollag, 2004). While players’ performance normally decreases with age, longer tenured players may bring other valuable skills in the game such as wisdom and mental toughness, which can be hardly found in less tenured players (Christie and Barling, 2010). That is why, in organizational settings, previous research has also used tenure as a proxy for individual’s expertise (Finkelstein, 1992; Zajac and Westphal, 1996; Krause et al., 2015). Finally, NBA salary schedules, which award longer tenured players with higher minimum salaries, corroborate the power given to these players. Hence, we used tenure in the league (instead of team tenure) as an indicator of power, with longer tenure indicating more power.

Third, the dominance (i.e., playing time in minutes) of each player in a given season (Ethiraj and Garg, 2012) as a proxy for structural power. Each NBA game lasts 48 min (excluding possible overtime). The more playing time a specific player gets, the less the remaining time for all his teammates. Hence, dominance denotes a control over the critical resource of playing time in the NBA context (i.e., higher values indicate individual dominance and lower values equality). Dominance may be considered as a proxy of player’s structural power because the more a player is in the court the more his direct control over other players (and team’s) resources.

Fourth, the number of games in which the player appears in the starting line-up as a proxy for prestige. Teams in our data set were comprised of 12 players each and played 82 games per season (except 1999 and 2012, which were shorter due to a lockout—max 50 and 66 games, respectively); thus, each player in each team could have started from 0 to 82 games each year. A player in the starting line-up usually gets heightened media attention (see, for instance, Favale, 2021), is more likely to prove his value, and is characterized as a “starter” (vs. non-starter), indicating his importance for the team decision makers (Christie and Barling, 2010). Hence, starters hold more informal power/prestige, which increases as they keep appearing to their team’s starting line-up.

To create the final power measure for each player, we made each indicator consistent to each other (i.e., measured in a similar scale) and relative to each team’s total resources (since each player is nested within a specific team with specific resources). For instance, Dwight Howard who played for Houston Rockets in 2015 had the highest salary in his team (i.e., $21.4 m), 11 years tenure in the league, 41 games as a starter, and 1.3 thousand minutes played. However, the maximum salary in the league (i.e., NBA) in 2015 was $23.5 m and his salary was ranked only fourth overall. This example indicates why player-level indicators have to be weighted in relation to each team and transformed to be consistent to each other.

Therefore, to weigh each indicator and compute the final power measure, we adapted well-established procedures used in previous research utilized for the NBA context (e.g., Christie and Barling, 2010). First, we divided each indicator by the team maximum value (e.g., *SALARY/SALARY*_team_max_) in order to determine the indicator strength for each player observation, ranging from 0 to 1. The indicators were then equally weighted and combined into a single power score with the following formula:


$$P_{i,k}=\left[\begin{array}{l} 1-\left(1-s\left({\mathrm{Salary}}_{i,k}\right)\right)\times \left(1-s\left({\mathrm{Tenure}}_{i,k}\right)\right)\\ \times \left(1-s\left({\mathrm{Games Started}}_{i,k}\right)\right)\\ \times \left(1-s\left({\mathrm{Dominance}}_{i,k}\right)\right) \end{array}\right]/P_{\mathrm{team}\_\max,k}$$

where P_i,k_ is the power score for player observation *i*, s(Salary_i,k_) is the weighted power indicator strength of player observation *i*’s salary, s(Tenure_i,k_) is the weighted power indicator strength for tenure in the league (in years) by player observation *i*, s(Games Started_i,k_) is the weighted power indicator strength for the number of games started by player observation *i*, s(Dominance_i,k_) is the weighted power indicator strength for the playing time (in minutes) by player observation *i*, and P_team_max,k_ is the maximum power score in team *k*.

#### Independent Variables

Player’s *competence* (i.e., contribution to team wins) was measured by win shares standardized per 48 min played (WS48), a measure developed by Oliver (2004) and previously used in papers using NBA as a research context (e.g., Hoffer and Freidel, 2014; Radzevick, 2016).

Following Christie and Barling (2010), we used player transgressions to measure *uncooperative behavior*. Transgressions included suspensions from play (i.e., NBA commissioner decides a suspension from play for on-court incidents, conduct that does not conform to standards of fair play, conduct that does not comply with federal or state laws, and conduct that is detrimental to the game of basketball or the league) and ejections from a game (i.e., when a player accumulates two technical fouls of an unsportsmanlike nature in the same game). Unlike most personal fouls (or a single technical foul), which also penalize players for breaking rules, “…transgressions are not the result of strategic play or a focus on team-oriented outcomes; they are inconsistent with team goals and reflect unproductive, uncooperative, and non-instrumental team behavior…” (see also Christie and Barling, 2010, p. 924).

Finally, we assessed a *team’s performance* with two measures: (a) following Zhang (2017), we computed *team competitiveness* as the team’s average win-loss record in the past three seasons, and (b) following Ethiraj and Garg (2012), we calculated *team playoffs* as the sum of the number of times a team reached the playoffs in the past 3 years.

#### Control Variables

Following scholars utilizing NBA as a research context (e.g., Christie and Barling, 2010; Ethiraj and Garg, 2012; Ertug and Castellucci, 2013), we controlled for the most common individual- and team-level characteristics that may influence power and its persistence in a team. In terms of individual-level characteristics, we controlled for player’s *age, race* (0 = black; 1 = other)*, height*, *weight*, *ethnicity*, *draft position,* and *playing position* (0 = center, forward; 1 = guard). *Physical health* was measured through player absences (i.e., number of occurrences of games missed) due to injury or illness divided by the total number of games in which a player appeared (similarly to Christie and Barling, 2010). We also controlled for *team mean power* and *pay dispersion* in our analyses (Christie and Barling, 2010). Finally, we controlled for *coach team tenure*, *coach NBA tenure* (in the league), and *coach performance* (Zhang, 2017). The latter was measured as the average coach win-loss record in the past 3 years (from 1994 and on).

#### Econometric Estimations

The composite power measure is more likely an ordinal variable rather than a cardinal or ratio-scale variable for three reasons. First, an ordinal variable is allowing to order individuals with respect to the characteristic of interest, but with the differences between individuals being incomprehensible. Instead, in cardinal or ratio-scale measures differences are meaningful (e.g., have a natural interpretation). For example, imagine player 1 in team X who has power of 0.80 (maximum in team X) and player 2 in team X who has power 0.76. This means that player 1 is more powerful than player 2 in team X with a power difference of 0.04 (i.e., P_Player1_- P_Player2_). Similarly, and throughout the dataset, compare player 1 with player 3 from team Y, who has power 0.89 (maximum in team Y). Player 3 is more powerful than player 1, both are the most powerful individuals in their teams, but the difference of their power (i.e., 0.09) is not meaningful in the sense that it does not provide evidence on how much powerful is player 3 in team Y as compared to player 1 in team X. This is because they are nested within different teams and power is relative to the team each player belongs to (even after controlling for team mean power). Hence, based on this example, the power measure clearly indicates the power of a specific player but the exact power differences between, e.g., two powerful players are not easily comprehensible. Furthermore, since our aim is to explore power persistence at the individual level, the natural interpretation of the power difference, e.g., between players 1 and 3 is not imperative. Second, recent theoretical evidence on individuals’ power in organizational settings reinforces our statement that power seems to be interpreted and perceived as an ordinal variable, falling into three distinct categories (i.e., high power, middle power, and low power), especially when trying to examine the transitions between them (Anicich and Hirsh, 2017). Third, the composite power variable follows an asymmetric (non-normal) distribution with spikes (k-density = 0.039). According to (DeCoster et al., 2009, p. 4), for variables with irregular distributions “…it is possible that there are specific types of distributions in which dichotomized indicators provide better representations of the underlying constructs than the observed continuous indicators….” Overall, and due to the above-mentioned reasons, we have decided to recode the composite power variable to a categorical one specifying three distinct power categories: low power, middle power, and high power. Specifically, we used the “cut” command in Stata to form three quantiles with approximately the same number of observations per category (i.e., tertiles).

Utilizing the panel nature of our data, random effects ordered probit specifications were mainly applied to explicitly account for individual’s time-invariant unobserved heterogeneity, such as race, ethnicity, gender, height, weight, and personality traits. In all our analyses, the independent variables were lagged 1 year behind the dependent variable. In this context, an underlying latent variable $F_{it}^{*}$ could be modeled as a vector of observed and unobserved individual characteristics. In particular, for each individual *i*, observed over a number of years *t* = 1, ..., 19, we specified


(1) $$F_{it}^{*}=x_{itk}^{\prime }\beta_{k}+u_{it}$$

where $x_{itk}^{\prime }$ is a vector of *k* observed individual characteristics, $u_{it}$ is a composite error term consisting of *α*_i_, which is a time-invariant component capturing unobserved individual-specific heterogeneity, and *ε*_it_ is a transitory random error term. We assumed that both *α*_i_ and *ε*_it_ were normally distributed, orthogonal to each other, and orthogonal to the observed characteristics. In our data, the latent outcome $F_{it}^{*}$ was not observed. However, it was related to an array of observed alternative power outcomes defined for the needs of our study using the power categories of the following form


(2) $$F_{it}=j\;\;\mathrm{if}\;\;\mu_{j-1}<F_{it}^{*}\leq \mu_{j},\;j=1,2,3$$

where it was assumed for the boundary parameters that $\mu_{0}=-\infty$, $\mu_{j}\leq \mu_{j+1}$, and $\mu_{m}=\infty$. Maximum likelihood estimation of model (2), alongside the Gaussian quadrature procedure, will produce estimates of the unknown boundary parameters and βs (Greene, 2003). The presence of unobserved heterogeneity requires rejection of the hypothesis that the variance due to unobserved heterogeneity, i.e., $\lambda =\sigma_{a}^{2}/\left(\sigma_{a}^{2}+\sigma_{\varepsilon }^{2}\right)$, is equal to zero.

Given the assumptions of normality, the probability of being classified in one of the power categories (*j*=1, 2, 3) is given by


(3) $$P\left(F_{it}=j|X\right)=\Phi \left(\mu_{J}-x_{itk}^{\prime }\beta_{k}-a_{i}\right)-\Phi \left(\mu_{j-1}-x_{itk}^{\prime }\beta_{k}-a_{i}\right)$$

where Φ(.) stands for the normal distribution function. For estimation purposes, it is needed to adopt a conventional normalization setting for the intercept terms $\beta_{0}=0$ (alternatively, it could be assumed that $\mu_{1}=0$).

The persistence of power categories could be typically tested by including lagged power states in equation (1). Thus,


(4) $$F_{it}^{*}=F_{it-1}^{\prime }\gamma +x_{itk}^{\prime }\beta_{k}+\alpha_{i}+\varepsilon_{it}$$

where $F_{it-1}$ is a vector of indicators for the individual’s power state in the previous year and *γ* represents the correspondent parameters to be estimated. The existence of state dependence requires the rejection of the null hypothesis that *γ*=0, while simultaneously controlling for unobserved heterogeneity.

However, the proposed approach requires that the beginning of the process *F*_it−1_ is uncorrelated with *a*_i_ (i.e., the “initial conditions” problem). This assumption, of the exogeneity of initial conditions, is valid when the errors are serially independent, and the first observation is the true initial outcome of the process. In our context, since both assumptions were unlikely to hold, the obtained results may be attributed to inconsistent estimators, meaning that the true state dependence could be overestimated (Heckman, 1981). Wooldridge (2005) suggested an approach to deal with this problem by including *F*_i1_ as an additional variable in equation (4). This conditional maximum likelihood (CML) approach implies that all outcomes *F*_i2_,…*F*_iT_ are conditional on the initial value *F*_i1_. Moreover, since the random effects models do not allow the observed regressors to be correlated with the individual effect (*a*_i_), we relaxed this assumption by using the time-averages of the time-varying covariates (Mundlak, 1978; Chamberlain, 1984). Thus, the unobserved individual-specific heterogeneity was specified by


(5) $$a_{i}=a_{0}+F_{i1}^{\prime }a_{1}+{\bar{x}}_{i\mu }\delta_{\mu }+v_{i}$$

where $F_{i1}^{\prime }$ is the vector of initial period power, ${\bar{x}}_{i\mu }$ stands for the time-average of the time-varying covariates (*μ* out of *k* variables) included in $x_{itk}^{\prime }$, and *v*_i_ is the independent normally distributed error term. Substituting equation (5) into equation (4) gives the dynamic correlated random effects ordered probit model to be estimated.

It should be noted that although Wooldridge’s specifications have been extensively applied in previous empirical studies that modeled dynamic ordered or discrete choices (Contoyannis and Li, 2011), the consistency of the estimator could be sensitive to misspecification of the individual effects since a model for the unobserved effects is specified (Contoyannis and Li, 2011). However, the alternative of applying the theoretically more robust fixed effects estimators (since it avoids the initial conditions problem and the specification of the relationship between individual’s effects and the covariates) suffers from the incidental parameter problem. Indeed, there are no general solutions for non-linear models with fixed effects, and where a specific solution is available it is not root-N-consistent.

## Results

Table 1 presents descriptive statistics and correlations for the study variables, most of which were significantly correlated with power. Table 2 reports the probability of transitioning from one power state to another. Results in the diagonal indicate that the probability of transitioning from a power state to another one was, generally, low.

**Table 1:** Descriptive statistics and correlations.

Variables	Mean	SD	1	2	3	4	5	6	7	8	9	10	11	12	13	14	15	16	17
1. Race^a^	0.22	0.41																	
2. Age	27.85	4.36	−0.00																
3. Height	79.11	3.67	0.27^**^	−0.03^**^															
4. Weight	220.47	28.13	0.20^**^	−0.09^**^	0.82^**^														
5. Ethnicity	64.38	15.76	−0.26^**^	0.05^**^	−0.18^**^	−0.16^**^													
6. Draft position	21.33	16.05	0.10^**^	−0.03^**^	−0.09^**^	−0.06^**^	−0.05^**^												
7. Playing position^a^	0.58	0.49	−0.20^**^	−0.03^**^	−0.73^**^	0.72^**^	0.13^**^	0.03^*^											
8. Uncooperative behavior	0.01	0.07	−0.02	0.00	0.00	0.00	0.01	−0.01	0.00										
9. Competence	0.07	0.09	0.02	0.09^**^	0.06^**^	0.07^**^	−0.01	−0.16^**^	−0.08^**^	−0.01									
10. Physical health	1.52	8.24	0.01	−0.03^**^	0.06^**^	0.49^**^	0.00	0.01	−0.06^**^	0.00	−0.16^**^								
11. Team competitiveness	1.15	0.63	0.03^*^	0.19^**^	0.00	−0.02^*^	−0.07^**^	0.03^*^	0.02	0.00	0.20^**^	−0.03^*^							
12. Team in playoffs	1.58	1.18	0.00	0.18^**^	0.00	−0.01	−0.05^**^	0.03^*^	0.01	0.00	0.17^**^	−0.02	0.80^**^						
13. Team mean power	0.45	0.06	−0.01	0.01	0.00	0.00	0.02	−0.06^**^	0.00	0.02	0.01	0.00	−0.17^**^	−0.18^**^					
14. Pay dispersion	142.23	263.49	−0.01	0.00	0.00	0.00	−0.02^*^	0.02	0.00	0.00	−0.04^**^	−0.01	−0.01	−0.01	−0.23^**^				
15. Coach team tenure	3.63	3.47	0.05^**^	0.05^**^	0.00	0.01	−0.08^**^	0.07^**^	0.00	0.00	0.08^**^	−0.02	0.42^**^	0.34^**^	−0.12^**^	0.06^**^			
16. Coach NBA tenure	8.61	7.13	0.00	0.03^**^	0.00	0.00	−0.04^**^	0.01	0.00	0.00	0.03^**^	0.00	0.19^**^	0.18^**^	−0.02^*^	0.07^**^	0.48^**^		
17. Coach performance	1.18	0.65	0.02^*^	0.17^**^	0.00	−0.02	−0.07^**^	0.02	0.02	0.00	0.20^**^	−0.02	0.88^**^	0.69^**^	−0.15^**^	0.00	0.39^**^	0.18^**^	
18. Player power	0.45	0.29	−0.06^**^	0.45^**^	0.00	−0.01	0.04^**^	−0.38^**^	0.00	0.02	0.37^**^	−0.14^**^	−0.02^*^	−0.02^*^	0.21^**^	−0.06^**^	−0.03^**^	−0.02	−0.02

*n = 8383*.

a *Dummy variable*.

* *p < 0.05*.

** *p < 0.01*.

**Table 2:** Transition probabilities for power categories.

Previous year	Current year		
	Low power	Middle power	High power
Low power	65.46	29.65	4.89
Middle power	15.12	57.81	27.06
High power	1.69	22.87	75.44

*n = 8383. Unbalanced panel of NBA player-years ranging from 1997 to 2015*.

Table 3 presents the coefficients from the pooled order probit, the random effects ordered probit, and the random effects ordered probit with Wooldridge’s corrections (from now on Wooldridge’s dynamic ordered probit) models. The results were, in most cases, similar with respect to the sign of the coefficients. However, some differences on statistical significance can be observed when we applied dynamic specifications that account for the unobserved individual heterogeneity and the initial conditions. Although pooled specifications provide useful descriptive models of the ordered power states under investigation, dynamic specifications are preferred because they also account for the unobserved time-invariant heterogeneity. Results from the quasi-likelihood-ratio statistics (in the lower part of Table 3) also confirmed the superiority of the random effects specifications compared to the pooled ordered probit model and of Wooldridge’s dynamic ordered probit model compared to random effects ordered probit specifications.

**Table 3:** Coefficients of the pooled ordered probit, random effects ordered probit, and Wooldridge’s dynamic ordered probit models on the probability of power.

Independent variables	Pooled ordered probit	Random effects ordered probit	Wooldridge’s dynamic ordered probit
Middle power at period *t* = 1	–	–	0.212^***^ (0.064)
High power at period *t* = 1	–	–	0.421^***^ (0.088)
Middle power at period *t*−1	–	–	0.683^***^ (0.069)
High power at period t−1	–	–	1.236^***^ (0.098)
Race	−0.225^***^ (0.041)	−0.220^***^ (0.085)	−0.127^**^ (0.058)
Age	0.173^***^ (0.056)	0.556^***^ (0.102)	0.285^***^ (0.087)
Height	−0.000 (0.001)	−0.014 (0.017)	−0.003 (0.013)
Weight	0.002^*^ (0.001)	0.002 (0.002)	0.002 (0.002)
Ethnicity	−0.000 (0.001)	0.004^*^ (0 0.002)	0.004^**^ (0 0.002)
Draft position	−0.021^***^ (0.001)	−0.025^***^ (0.003)	−0.013^***^ (0.002)
Playing position	0.225^***^ (0.053)	0.132 (0.087)	0.133 (0.069)
Uncooperative behavior	0.055 (0.119)	−0.472^***^ (0.143)	−0.517^***^ (0.145)
Competence	4.709^***^ (0.614)	2.422^***^ (0 0.512)	1.755^***^ (0 0.445)
Physical health	−0.000 (0.009)	−0.000 (0.007)	0.007 (0.006)
Team competitiveness	−0.266^***^ (0.067)	−0.408^***^ (0.088)	−0.287^***^ (0.078)
Team in playoffs	−0.096^***^ (0.024)	−0.119^***^ (0.031)	−0.107^***^ (0.028)
Team mean power	1.534^***^ (0.305)	1.796^***^ (0.366)	1.837^***^ (0 0.337)
Pay dispersion	0.000 (0.000)	−0.000 (0.000)	−0.000 (0.000)
Coach team tenure	0.006 (0.005)	0.012 (0.008)	0.006 (0.006)
Coach league tenure	−0.000 (0.003)	−0.005 (0.004)	−0.002 (0.003)
Coach performance	0.001 (0.005)	−0.006 (0.065)	−0.014 (0.060)
Cut 1	−0.245 (1.062)	−3.622 (2.516)	−1.543 (1.794)
Cut 2	1.191 (1.062)	−1.656 (2.517)	0.369 (1.795)
ICC	–	0.381	0.138
Log- likelihood	−4510.011	−3921.060	−3762.307
Wald test (Ho: Cut1-Cut2)	1600.28^***^	1189.74^***^	2073.37^***^
Quasi-LR-test	–	512.17^***^	44.57^***^
Sample size	5601	5601	5601

*Standard errors in parentheses. The coefficients of the means of the independent variables and year dummies are not presented in the table but were included in the analyses of random effects and Wooldridge’s dynamic models*; ^*^p < 0.10; ^**^p < 0.05; ^***^p < 0.01.

Results from the random effects specification showed that higher competence, being African American, and having a better position in the draft were positively associated with the probability of achieving a state of high power. On the other hand, being uncooperative, being non-black (e.g., white or Asian), and playing for a better or more competitive team negatively affected the probability of achieving a state of high power. The proportion of the model’s variance due to the unobserved heterogeneity was about 38% in the random effect model (lowest part of Table 3). Wald tests on the null hypothesis of the equality of the boundary parameters rejected the null, indicating that the three power categories should not be collapsed and therefore were properly identified.

Because the estimated coefficients of the pooled and the random effects ordered probit models did not represent the magnitude of the association between power categories and its correlates, marginal effects for each of the three power states (low, middle, and high) should be estimated. Tables 4 and 5 present the corresponding marginal effects on the probability of the three power categories in pooled and random effects ordered probit specifications.

**Table 4:** Marginal effects of the pooled ordered probit model on the probability of power.

Independent variables	Low power	Middle power	High power
Race	0.048^***^	0.012^***^	−0.061^***^
Age	0.037^***^	0.009^***^	−0.046^***^
Height	−0.000	−0.000	0.000
Weight	−0.001^*^	−0.001^*^	0.001^***^
Ethnicity	0.000	0.000	−0.000
Draft position	0.004^***^	0.001^***^	−0.006^***^
Playing position	−0.048^***^	−0.012^***^	0.060^***^
Uncooperative behavior	−0.012	−0.003	0.144
Competence	−1.006^***^	−0.261^***^	1.267^***^
Physical health	0.000	0.000	−0.000
Team competitiveness	0.057^***^	0.015^***^	−0.071^***^
Team in playoffs	0.021^***^	0.005^***^	−0.026^***^
Team mean power	−0.327^***^	−0.085^***^	0.413^***^
Pay dispersion	−0.000	−0.000	0.000
Coach team tenure	−0.001	−0.000	0.002
Coach league tenure	0.000	0.000	−0.000
Coach Performance	−0.000	−0.000	0.000

^*^p < 0.10; ^**^p < 0.05; ^***^p < 0.01.

**Table 5:** Marginal effects of the random effects ordered probit model on the probability of power^a^.

Independent variables	Low power	Middle power	High power
Race	0.034^***^	0.031^***^	−0.065^***^
Age	−0.079^***^	−0.087^***^	0.167^***^
Height	0.002	0.002	−0.004
Weight	−0.000	−0.000	0.000
Ethnicity	−0.001^*^	−0.001^*^	0.001^*^
Draft position	0.004^***^	0.004^***^	−0.007^***^
Playing position	−0.019	−0.020	0.039
Uncooperative behavior	0.068^***^	0.074^***^	−0.142^***^
Competence	−0.348^***^	−0.379^***^	0.727^***^
Physical health	0.000	0.000	−0.000
Team competitiveness	0.059^***^	0.063^***^	−0.122^***^
Team in playoffs	0.017^***^	0.019^***^	−0.036^***^
Team mean power	−0.258^***^	−0.281^***^	0.539^***^
Pay dispersion	0.000	0.000	−0.000
Coach team tenure	−0.002	−0.002	0.003
Coach league tenure	0.000	0.000	−0.001
Coach Performance	0.000	0.000	−0.001

a Source: Authors’ own calculations. ^*^p < 0.10; ^**^p < 0.05; ^***^p < 0.01.

Next, we present results from the dynamic random effects model when state dependence and initial conditions are considered, namely, the Wooldridge dynamic ordered probit model (Tables 3 and 6). The estimated coefficients from the lagged categories of the dependent variable were large in magnitude and highly significant. There was a gradient across the effects of the lagged power states as individuals moved from a previous state of low power to a state of high power. Additionally, the estimated coefficients for initial power were also highly significant and revealed a similar positive gradient. The results in Tables 3 (right part) and 6 indicated that power outcomes were characterized by significant genuine state dependence (i.e., non-spurious correlations but genuine effects), thus confirming Hypothesis 1. Initial conditions also played a significant role on later power states. Furthermore, the observed substantial reduction on the model’s variance due to the unobserved heterogeneity compared to the pooled (non-dynamic) and the random effects specifications confirmed the importance of accounting for state dependence in our models (Table 3 lowest part). Indeed, the proportion of the variance attributed to unobserved heterogeneity reduced to 13.8% in Wooldridge’s dynamic model (a reduction of 24.3%) in comparison to the random effects model.

**Table 6:** Marginal effects of the Wooldridge’s dynamic ordered probit model on the probability of power^a^.

Independent variables	Low power	Middle power	High power
Middle power at period *t* = 1	−0.027^***^	0.048^***^	0.075^***^
High power at period *t* = 1	−0.048^***^	0.102^***^	0.150^***^
Middle power at period *t*−1	−0.081^***^	0.159^***^	0.241^***^
High power at period *t*−1	−0.148^***^	0.276^***^	0.424^***^
Race	0.018^**^	0.026^**^	−0.044^**^
Age	−0.038^***^	−0.061^***^	0.099^***^
Height	0.000	0.001	−0.001
Weight	−0.000	−0.000	0.001
Ethnicity	−0.001^**^	−0.001^**^	0.001^**^
Draft position	0.002^***^	0.003^***^	−0.004^***^
Playing position	−0.018	−0.028	0.046
Uncooperative behavior	0.069^***^	0.111^***^	−0.180^***^
Competence	−0.274^***^	−0.378^***^	0.612^***^
Physical health	−0.001	−0.002	0.002
Team competitiveness	0.038^***^	0.062^***^	−0.100^***^
Team in playoffs	0.014^***^	0.023^***^	−0.037^***^
Team mean power	−0.245^***^	−0.396^***^	0.641^***^
Pay dispersion	0.001	0.001	−0.000
Coach team tenure	−0.001	−0.001	0.002
Coach league tenure	0.000	0.000	−0.001
Coach Performance	0.002	0.003	−0.005

a Source: Authors’ own calculations. ^*^p < 0.10; ^**^p < 0.05; ^***^p < 0.01.

Most of the effects of the covariates in Wooldridge’s dynamic model followed a similar direction as those in the random effects model. Specifically, higher competence was positively associated with the probability of achieving a state of high power, thus confirming Hypothesis 2. Also, being older, African American, and having a better position in the draft increased the probability of achieving a high-power state. On the other hand, being uncooperative and playing for a better performing or more competitive team negatively affected the probability of sustaining a high-power state, therefore confirming Hypothesis 3 and 4.

To understand the magnitude of the association between power states and its correlates, marginal effects are presented in Table 6. Specifically, we present the marginal effects on the probability of being classified in low, middle, and high-power states. Players that were previously (*t*−1) in a state of high power have a probability of 42.4% of remaining in the high-power state in the later period (*t = 1*) compared to those classified as low power (reference category). Those classified as middle power at *t*−1 have a 24.1% higher probability of being classified as high power in period *t = 1*. With respect to the initial conditions, being classified as high (middle) power increased the likelihood of later high-power states for about 15 (7.5) per cent, compared to the reference category.

Marginal effects on the other covariates of the dynamic specifications showed that players who had higher contribution to team wins, were African Americans, and had a better position in the draft experienced a higher probability of being classified as high power of about 61.2, 1.8, 0.1, and 0.4 per cent, respectively. Lower probabilities of achieving a high-power state were experienced by players who behaved uncooperatively (18%), who played for a better (3.7%) or more competitive team (10%), and were non-black (e.g., white or Asian; 4.4%).

Overall, the obtained results showed that although unobserved heterogeneity (such as genes or individual’s traits) plays an important role in explaining the observed persistence across power states, genuine state dependence (correlation between current and past power states) was also a significant contributor. The conditional role of the observed heterogeneity in explaining power variations was also evident in the above analysis.

## Discussion

This study examined an important and timely topic in the power literature, namely, the dynamics of individuals’ power in teams over time (Anderson and Brion, 2014; Sturm and Antonakis, 2015). Reconciling the premises of the functional (Magee and Galinsky, 2008; Tarakci et al., 2016) and conflict perspectives (Greer and van Kleef, 2010; Sturm and Antonakis, 2015; Greer et al., 2017) on power, we proposed and found that individuals’ power is comprised of *both* static and dynamic elements.

Hypothesis 1 focused on whether power persists over time, thus testing the claims of the functional account of power, which proposes that power is self-reinforcing in nature (Magee and Galinsky, 2008). Our longitudinal design allowed us to investigate the genuine state dependence of an earlier to a later state of power, and we showed that power indeed persisted over time. More specifically, we found that an individual who was high-power at *t*−1 was also more likely to be powerful at *t* = 1.

Hypotheses 2–4 focused on more dynamic elements of power, thus testing the conflict perspective of power, which suggests that power structures can be changed (Greer et al., 2017). Specifically, we investigated three “key” factors that affected power over and above its persistence. Confirming Hypothesis 2, we provided empirical evidence that competence positively affected power beyond its self-reinforcing nature. This result extends past research supporting a strong and positive link between competence and power (Treadway et al., 2013; Anderson and Brion, 2014; Guinote, 2017).

Furthermore, confirming Hypothesis 3, individuals’ uncooperative behavior was found to negatively affect power above and beyond its persistence, revealing that behaving uncooperatively in highly interdependent teams may lead to individuals’ loss of power. Uncooperative behaviors typically refer to actions that benefit oneself at the expense of others (Bendersky and Shah, 2012; Mead and Maner, 2012). Previously, scholars noted that powerful individuals may behave more selfishly (Gruenfeld et al., 2008) and use their power to violate social norms and serve themselves to the detriment of common good (Handgraaf et al., 2008; Bendahan et al., 2015). This study’s results, however, demonstrated that uncooperative behaviors may not only hurt the team but also may not bring the desired gains for oneself; on the contrary, they may hinder one’s power in the team.

Additionally, and in line with Hypothesis 4, this study showed that team performance was detrimental for individual’s power over and above its persistence. Often, individuals who strive for power also attempt to join a high-performing team, as a means to gain power (Sheridan et al., 1990). However, this might not be the right strategy. In high-performing teams, competition between players is higher and chances to gain power may thus decrease. Indeed, this study’s findings suggest that high-level competition makes individuals’ efforts to acquire and maintain power challenging (Sutton and Galunic, 1996; Anderson and Brion, 2014). This result extends previous research, which suggested that the co-existence of high-power individuals undermine team collaboration and performance in teams requiring high levels of coordination (Hildreth and Anderson, 2016), by showing that individual-level power is also hindered in highly competitive and interdependent teams.

In sum, the above-mentioned results give answers to recent calls for investigating the factors affecting the maintenance and loss of power in team settings in the long-term (Anderson and Brion, 2014; Sturm and Antonakis, 2015). Specifically, our analyses disentangled the properties of power (i.e., self-reinforcing nature) from its antecedents and incorporated the fact that some individuals’ power changes dynamically and others’ remain unchanged at the same time.

### Implications for Practice

The outcomes of this study provide important implications for the configuration of power in teams as our results indicate the specific factors affecting individuals’ power. Managers may use this knowledge to effectively handle team power struggles by increasing or decreasing individual team members’ power. Specifically, we showed that in highly competitive teams, individuals acquired power (i.e., through their competence) but, at the same time, they also lost power (i.e., due to the high team competitiveness). Managers could utilize these findings to increase (or decrease) power disparity within their teams (Tarakci et al., 2016). For instance, if managers wish to develop flat hierarchies (e.g., in agile teams), they may try to bring people together in a team that have comparable competence. Adjusting power differences within teams has been shown to improve teamwork, team learning (Chen and Garg, 2018), and team performance (Greer et al., 2017).

In terms of implications for individuals in team settings, our results may explain why highly competent individuals sometimes lose their stardom (i.e., because of individual or team-level factors such as uncooperative behavior or team performance) or why their perceived value does not always lead to the sustainability of their stardom (Call et al., 2015; Kehoe et al., 2018). Hence, high-power individuals may use our findings to sustain their hierarchical position in teams.

### Limitations and Future Research

The current study has some limitations that create opportunities for future research. While the NBA context provided us with objective and longitudinal data, one may argue whether our results can be generalized to other business organizations. Generalizations should always be done with caution; however, it is important to note that past research found that the NBA context is valid and the results can be compared to those in real business settings (see also Ethiraj and Garg, 2012; Ertug and Castellucci, 2013; Smith and Hou, 2014; Zhang, 2017).

To test our specific predictions, the NBA context was in fact an asset. One reason is that in order to test our predictions, we needed teams to be highly interdependent and individuals’ power may be largely related to their peers’ inputs and outputs. This is clearly the case in the NBA context. In other organizational contexts where individuals’ interdependence is low (such as information technology teams or mutual funds companies), the effects of certain covariates (i.e., team performance) may be less pronounced. Also, the fact that in NBA teams cross-memberships do not exist, helped in disentangling the effects of the static and dynamic elements of individuals’ power. However, future research should investigate the consistency of our results in different types of teams operating under different levels of reciprocal interdependence.

A related concern refers to our measure of individuals’ power. Specifically, our measure calculates the objective power an individual has based on the resources he controls. Future research could validate this measure through a survey/perceptual study, which would benefit from direct feedback from players. This study could also examine whether individuals actualize or feel this power (e.g., Tost, 2015) and how felt (or not) power affects the factors influencing its acquisition and loss.

Furthermore, and based on the growing interest regarding metrics- and analytics-based performance and productivity (Harrower, 2019; Wingard, 2019; Nemteanu et al., 2021), future research could examine the effects of algorithmic decision making on individuals’ power dynamics in teams. Lastly, hiring algorithms, the commodification of reified persona, and the dynamics of individuals’ power in teams could present another fruitful avenue for future research (Pera, 2019; Sion, 2019; Nemteanu and Dabija, 2021).

### Conclusion

To reconcile the functional and conflict theories of power, this study tested a model in which both static and dynamic elements of power were present. Specifically, we found that individuals’ power persists over time, which is an assertion related to the functional theory of power. Second, and to go above and beyond power persistence, we relied on conflict theory of power to suggest there are a few “key” factors that influence the maintenance and loss of power in teams. Our results indicated that individuals’ competence positively influenced power while individuals’ uncooperative behavior and team performance negatively impacted power above and beyond its persistence. We discussed our findings in relation to managing power struggles in teams.

## Data Availability Statement

The raw data supporting the conclusions of this article will be made available by the authors, without undue reservation.

## Author Contributions

CM contributed to the research idea, collected the data, analyzed the data, and wrote the manuscript. PS contributed to the research idea and wrote the manuscript. All authors contributed to the article and approved the submitted version.

## Conflict of Interest

The authors declare that the research was conducted in the absence of any commercial or financial relationships that could be construed as a potential conflict of interest.

## Publisher’s Note

All claims expressed in this article are solely those of the authors and do not necessarily represent those of their affiliated organizations, or those of the publisher, the editors and the reviewers. Any product that may be evaluated in this article, or claim that may be made by its manufacturer, is not guaranteed or endorsed by the publisher.

## Footnotes

^1^ http://www.basketball-reference.com

^2^ http://www.eskimo.com/~pbender/

^3^ http://www.dougstats.com

^4^ http://prosportstransactions.com/

^5^ https://sites.google.com/site/rodswebpages/codes

^6^ https://www.forbes.com
